# Inhibition of the β-carbonic anhydrase from the protozoan pathogen *Trichomonas vaginalis* with sulphonamides

**DOI:** 10.1080/14756366.2020.1863958

**Published:** 2020-12-28

**Authors:** Linda J. Urbański, Andrea Angeli, Vesa P. Hytönen, Anna Di Fiore, Giuseppina De Simone, Seppo Parkkila, Claudiu T. Supuran

**Affiliations:** a Faculty of Medicine and Health Technology, Tampere University, Tampere, Finland; b Neurofarba Department, Sezione di Chimica Farmaceutica e Nutraceutica, Università degli Studi di Firenze, Firenze, Italy; c Fimlab Ltd, Tampere, Finland; d Institute of Biostructures and Bioimaging of the National Research Council, Naples, Italy

**Keywords:** Carbonic anhydrase, sulphonamide, inhibitor, *Trichomonas vaginalis*, trichomoniasis

## Abstract

Sulphonamides and their isosteres are classical inhibitors of the carbonic anhydrase (CAs, EC 4.2.1.1) metalloenzymes. The protozoan pathogen *Trichomonas vaginalis* encodes two such enzymes belonging to the β-class, TvaCA1 and TvaCA2. Here we report the first sulphonamide inhibition study of TvaCA1, with a series of simple aromatic/heterocyclic primary sulphonamides as well as with clinically approved/investigational drugs for a range of pathologies (diuretics, antiglaucoma, antiepileptic, antiobesity, and antitumor drugs). TvaCA1 was effectively inhibited by acetazolamide and ethoxzolamide, with K_I_s of 391 and 283 nM, respectively, whereas many other simple or clinically used sulphonamides were micromolar inhibitors or did not efficiently inhibit the enzyme. Finding more effective TvaCA1 inhibitors may constitute an innovative approach for fighting trichomoniasis, a sexually transmitted infection, caused by *T. vaginalis*.

## 1. Introduction

*Trichomonas vaginalis* is a protozoan parasite responsible for trichomoniasis, one of the most frequent non-viral sexually transmitted diseases in humans^1^^,^^2^. Treatment of this disease remains almost exclusively based on just one class of drugs, 5-nitroimidazoles (with two available agents, metronidazole and tinidazole), and resistance to these agents is on the rise worldwide^3^^,^^4^. Trichomoniasis may cause a variety of symptoms, from mild to severe, but a large fraction (10–50%) of infected women show no symptoms, and 5–15% of cases may remain undetectable upon examination^1^^,^^2^. Furthermore, the majority of infected men are totally asymptomatic, making the diagnosis of this disease particularly challenging^1^^,^^2^. *T. vaginalis* infection may facilitate or worsen other critical pathologies, such as HIV-infection^5^ or even prostate cancer^6^. As a consequence, research on novel drug targets for fighting trichomoniasis has seen an increased interest^7–11^.

Carbonic anhydrases (CA, EC 4.2.1.1) have recently been identified as new potential targets for finding alternative drugs that may interfere with or abolish crucial steps in the life cycle of protozoan parasites^7–11^. Recently, we have characterised a β-class CA, TvaCA1, which is one of the two such enzymes found in the proteome of *T. vaginalis*^10^. The other one, TvaCA2, will be described shortly elsewhere. Like the other members of this metalloenzyme superfamily^12–15^, TvaCA1 catalyses the interconversion between CO_2_ and bicarbonate, also generating protons, and is probably a key element of the molecular machinery involved in the pH regulation and metabolism of the parasite. Mammalian hosts, including humans, have several genes encoding α-class CAs in their genomes, whereas they have no β-CA genes^12^. For this reason, β-CAs may represent an interesting target for finding anti-infectives with a novel mechanism of action^16^. Accordingly, Flaherty’s group^17^ recently reported that sulphonamide CA inhibitors (CAIs), structurally related to acetazolamide (AAZ), have potent anti-bacterial effects against pathogenic drug resistant bacteria (which normally contain α-, β-, γ- and/or ι-CAs)^18^^,^^19^. They demonstrated this effect in vancomycin-resistant enterococci, validating thus *in vivo* the bacterial CAs as drug targets^16–17^.

Based on these considerations and with the aim to identify new targets for the development of innovative drugs against trichomoniasis, we undertook a detailed study on TvaCA1. We previously determined the X-ray crystal structure of this enzyme^10^ and described its inhibition with a wide range of inorganic anions^11^. Here we continue this study reporting the inhibition of TvaCA1 with a series of simple aromatic/heterocyclic primary sulphonamides as well as with clinically approved/investigational such drugs for a range of pathologies.

## 2. Materials and methods

### 2.1. Chemistry

Sulphonamides **1–24** were either commercially available or prepared as reported earlier by our group^20^. Clinically used agents **AAZ–HCF** were the reagents from Sigma-Aldrich (Milan, Italy) with the highest purity available.

### 2.2. Enzymology

TvaCA1 was a recombinant enzyme obtained in-house as described earlier^10^. The enzyme was recombinantly produced in *E. coli* (OneShot^®^ BL21 Star™ (DE3) Chemically Competent Cells, #C601003, Thermo Fisher Scientific, Finland). The enzyme was purified using Ni^2+^-NTA agarose affinity chromatography resin (Macherey-Nagel GmbH Co., Germany). More detailed information concerning the recombinant protein production and its kinetic and structural characterisation can be found in our previous article^10^. The presence of the correct protein in the isolated protein fraction was confirmed by tandem mass spectrometry (MS/MS).

### 2.3. CA catalytic activity and inhibition assay

An Applied Photophysics stopped-flow instrument was used for assaying the CA catalysed CO_2_ hydration activity^21^. Phenol red (at a concentration of 0.2 mM) was used as an indicator, working at the absorbance maximum of 557 nm, with 10 − 20 mM HEPES (pH 7.5, for α-CAs) or TRIS (pH 8.3 for β-CAs) as buffers, and 20 mM NaClO_4_ (for maintaining constant the ionic strength), following the initial rates of the CA-catalysed CO_2_ hydration reaction for a period of 10–100 s. The CO_2_ concentrations ranged from 1.7 to 17 mM for the determination of the kinetic parameters and inhibition constants. For each inhibitor, at least six traces of the initial 5–10% of the reaction were used for determining the initial velocity. The uncatalyzed rates were determined in the same manner and subtracted from the total observed rates. Stock solutions of inhibitors (10 mM) and dilutions up to 0.01 nM were prepared in distilled-deionised water. Inhibitor and enzyme solutions of concentrations ranging between 5 and 12 nM were preincubated together for 15 min at room temperature prior to assay, in order to allow for the formation of the enzyme–inhibitor complex. The inhibition constants were obtained by non-linear least-squares methods using PRISM 3 as reported earlier^22^, and represent the mean from at least three different determinations.

## 3. Results and discussion

**Figure 1.**
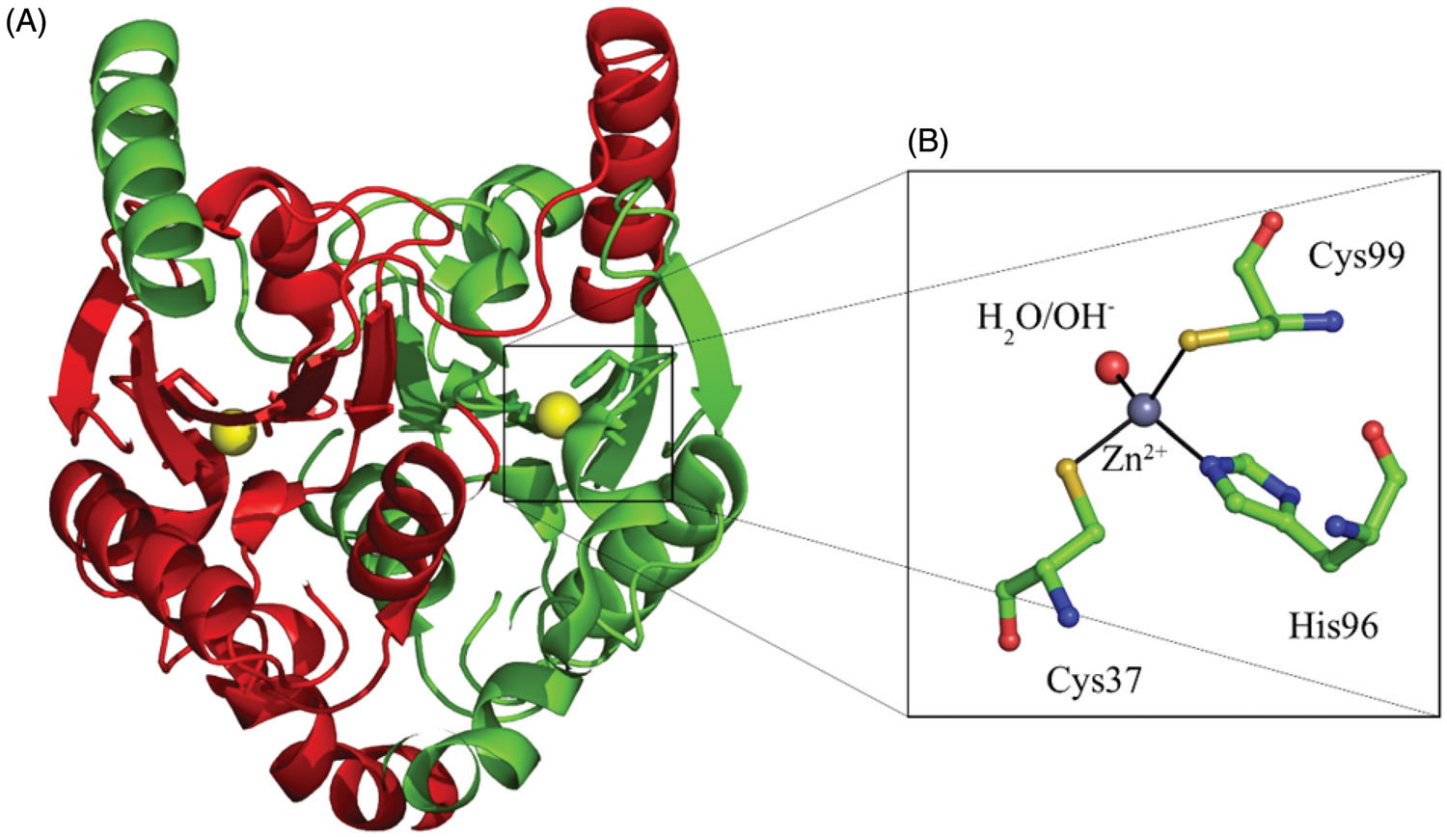
(A) TvaCA1 dimeric structure, with the two monomers shown in green and red, respectively^10^. (B) Active site of the enzyme, with the zinc ion (gray sphere) coordinated by two Cys, one His and one water molecule/hydroxide ion (shown in red). Residues numbering as described by Urbański et al.^10^.

TvaCA1 is a β-CA that has an open active site^23^, meaning that the water molecule/zinc hydroxide ion, acting as nucleophile in the catalytic cycle, is coordinated to the metal ion, as seen in Figure 1.

**Figure 2.**
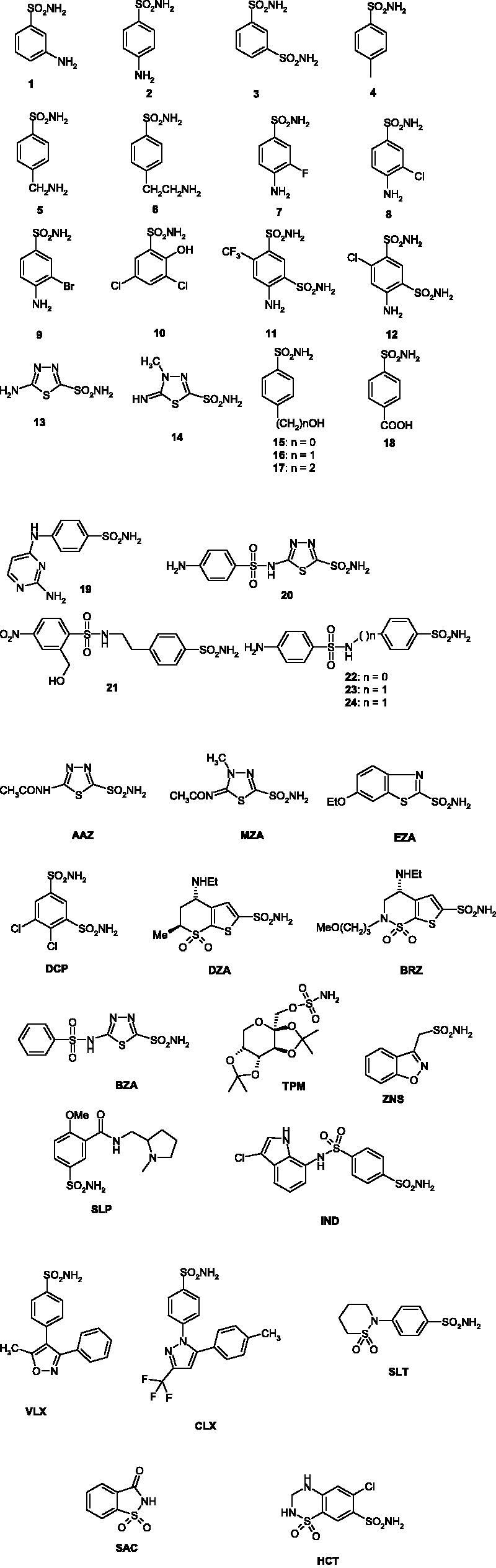
Sulphonamides **1–24** and clinically used agents **AAZ–HCT** investigated as TvaCA1 inhibitors.

As all β-CAs, TvaCA1 is a dimer with two narrow, channel-like active sites present in the molecule, as seen in Figure 1. Thus, this enzyme is rather different from the α-CAs present in the human host, which are generally monomeric enzymes with the zinc ion coordinated by three His residues and a water molecule, and possessing a rather ample active site where inhibitors and activators may bind^12–14^^,^^24^. Hence, we decided to investigate the inhibition of TvaCA1 with the main class of organic CA inhibitors, the primary sulphonamides, some of which are clinically used agents as diuretics, antiglaucoma, antiepileptic, antiobesity and antitumor agents^25–30^. In particular, a series of such compounds of types **1–24** and **AAZ–HCT** (Figure 2) were analysed.

Derivatives **1–24** and **AAZ–HCT** are simple aromatic/heterocyclic sulphonamides widely used as building blocks for obtaining new families of such pharmacological agents^20^^,^^22^, or they are clinically used drugs, such as acetazolamide **AAZ**, methazolamide **MZA**, ethoxzolamide **EZA** and dichlorophenamide **DCP**, which are the classical and systemically acting antiglaucoma CAIs. Dorzolamide **DZA** and brinzolamide **BRZ** are topically acting antiglaucoma agents, benzolamide **BZA** is an orphan drug belonging to this class of pharmacological agents, whereas topiramate **TPM**, zonisamide **ZNS** and sulthiame **SLT** are widely used antiepileptic drugs. Sulpiride **SLP** and indisulam **IND** were also shown by our group to belong to this class of pharmacological agents, together with the COX2 “selective” inhibitors celecoxib **CLX** and valdecoxib **VLX**. Saccharin and the diuretic hydrochlorothiazide **HCT** are also known to act as CAIs^25–30^.

The following structure–activity relationship (SAR) can be drawn from the inhibition data presented in Table 1, where hCA II inhibition is also given for comparison, considering that this is the most abundant human isoform^12^^,^^13^ and presumably the major off target enzyme when the use of CAI class anti-infectives are considered:

**Table 1.** Inhibition of human isoform hCA II, for comparison, and of the protozoan enzyme TvaCA1 with sulphonamides **1–24** and the clinically used drugs **AAZ–HCT**, measured by a CO_2_ hydrase, stopped-flow assay.^21^

Inhibitor/enzyme	K_I_^a^ (nM)	
	hCA II^b^	TvaCA1^c^
Class	α	β
**1**	300	3246
**2**	240	4742
**3**	8	3559
**4**	320	3599
**5**	170	>50,000
**6**	160	>50,000
**7**	60	4282
**8**	110	>50,000
**9**	40	>50,000
**10**	54	4536
**11**	63	>50,000
**12**	75	>50,000
**13**	60	1889
**14**	19	3987
**15**	80	2027
**16**	94	>50,000
**17**	125	>50,000
**18**	46	>50,000
**19**	33	4528
**20**	2	>50,000
**21**	11	3450
**22**	46	>50,000
**23**	33	>50,000
**24**	30	>50,000
**AAZ**	12	391
**MZA**	14	3827
**EZA**	8	283
**DCP**	38	>50,000
**DZA**	9	>50,000
**BRZ**	3	>50,000
**BZA**	9	>50,000
**TPM**	10	>50,000
**ZNS**	35	>50,000
**SLP**	40	>50,000
**IND**	15	>50,000
**VLX**	43	>50,000
**CLX**	21	>50,000
**SLT**	9	>50,000
**SAC**	5959	>50,000
**HCT**	290	>50,000

^a^ Errors in the range of 5–10% of the shown data, from 3 different assays.

^b^ Human recombinant isozyme, measured by stopped flow CO_2_ hydrase assay method.

^c^ Recombinant protozoan enzyme, measured by stopped flow CO_2_ hydrase assay method, this study.

(i) Only 14 of the 40 tested derivatives **1–24** and **AAZ–HCT** inhibited TvaCA1, while the remaining 26 were ineffective at concentrations up to 50 µM in the assay system. The ineffective group of compounds included the clinically used agents **DCP, DZA, BRZ, BZA, TPM, ZNS, SLP, IND, VLX, CLX, SLT, SAC** and **HCT**. One should note that apart from **SAC**, which is a secondary sulphonamide with a rather compact scaffold, the remaining ineffective derivatives possess rather bulky scaffolds and various substituents on which the sulphonamide or sulfamate moieties are appended. This may explain why these compounds do not access easily the narrow channel-like active site of this β-class enzyme, although they generally act as effective hCA II inhibitors. As mentioned above, the active site of the α-CAs is much wider compared to that of the β-CAs. Furthermore, some of the simple derivatives **1–24** were ineffective as TvaCA1 inhibitors including **5, 6, 8, 9, 11, 12, 16–18, 20,** and **22–24** (Table 1). These sulphonamides belong to heterogeneous classes, such as simple amino/hydroxyl-alkyl-substituted-benzenesulfonamides (**5, 6, 16, 17**), halogeno-sulfanilamides (**8** and **9**), 1,3-benzenedisulfonamides (**11** and **12**), and sulfonylated-sulphonamides with an elongated molecule (**20, 22–24**). As mentioned for the clinically used agents, some of these derivatives also possess rather bulky scaffolds that probably interfere with their efficient binding within the enzyme active site.

(ii) Sulphonamides **1–4, 7, 10, 13–15, 19, 21** and **MZA** were micromolar TvaCA1 inhibitors, with inhibition constants ranging between 1.889 and 4.742 µM (Table 1). From a structural point of view it may be observed that most of them incorporate compact, simple benzenesulfonamide/thiadiazole sulphonamide scaffolds with few or just one compact substituent (such as **1–4, 7, 13–15** and **MZA**). However, at least two of these derivatives, **19** and **21**, possess more complex scaffolds with elongated molecules which presumably are able to accommodate within the enzyme active site. Thus, it should be in principle possible to find or design even more effective inhibitors considering these two compounds as lead molecules.

(iii) The most effective TvaCA1 inhibitors were acetazolamide **AAZ** and ethoxzolamide **EZA**, with K_I_s of 391 and 283 nM, respectively. However, no inhibitor with an inhibition constant < 100 nM was detected for the *Trichomonas* enzyme.

## 4. Conclusions

We report the first inhibition study with sulphonamides of one of the β-CAs, TvaCA1, found in the protozoan parasite *T. vaginalis*. Only anion inhibitors and other small molecules were reported earlier to act as millimolar TvaCA1 inhibitors. Here we investigated 40 sulphonamides, some of which are clinically used drugs, for their inhibitory interaction with this enzyme. Only 16 of these agents showed inhibitory effects, most of them in the low micromolar range, whereas the most effective TvaCA1 inhibitors were acetazolamide **AAZ** and ethoxzolamide **EZA**, with K_I_s of 391 and 283 nM, respectively. Although no *in vivo/ex vivo* studies have been performed so far, inhibition of this protozoan enzyme may show anti-infective effects, as was reported for other protozoan species such as *Leishmania donovani chagasi*^31^ or *Trypanosoma cruzi*^32^, for which CAIs belonging to various classes showed potent anti-protozoan activity *in vivo*. Thus, effective TvaCA1 inhibitors may lead to the development of novel anti-infectives with a diverse mechanism of action.
